# Rapid Preparation of Electrocompetent *Listeria monocytogenes* and Enhancement of Transformation Efficiency with cAMP Supplementation^☆^

**DOI:** 10.1016/j.jfp.2026.100753

**Published:** 2026-05

**Authors:** A.V. Gutiérrez, N. Som, E. Smith, M. Diaz, M. Matthews, R.A. Kingsley, M. Gilmour

**Affiliations:** 1 Quadram Institute Bioscience, Norwich Research Park, Norwich, UK; 2 University of East Anglia, Norwich, UK; 3 Center For Microbial Interactions, Norwich, UK

**Keywords:** Agar‐lawn protocol, Competence, Cyclic AMP (cAMP), Defense‐antidefense systems, *L. monocytogenes*, Transformation

## Abstract

*L. monocytogenes* is a significant foodborne pathogen associated with serious health risks. The ability to genetically manipulate this bacterium is critical for understanding its pathogenic mechanisms and developing new interventions. However, low transformation efficiency and the absence of natural competence in *L. monocytogenes* present challenges for genetic studies. We optimized transformation by testing two isolates closely related to widely used laboratory reference strains F2365 and EGD-e (BL87-016 and BL91-025, respectively), and one CC121 isolate (BL87-028-B) that is ecologically relevant to food environments. Increasing the electroporation voltage from 10 to 11 kV/cm resulted in an increase in efficiency. In addition, supplementation with cyclic adenosine monophosphate (cAMP) prior to electroporation increased transformation efficiency in a dose-dependent manner, with an optimal concentration of 32 mM cAMP yielding increases of up to 377-fold. Recognizing the time constraints associated with liquid-based protocols, we developed a rapid agar-lawn method that simplified the workflow and reduced preparation time from approximately 10 h to under 3 h, though efficiencies remained lower than the gold standard method by Monk et al. (2008). The rapid agar-lawn protocol was tested in a panel of 66 *L. monocytogenes* isolates, with 83% of isolates showing transformability. Transformability showed no statistical correlation between lineage or mobile genetic elements (MGEs). Cooccurrence network analysis of defense and antidefense systems uncovered a higher incidence of interconnected defense repertoires in transformable isolates, suggesting adaptive immune architectures that facilitate DNA uptake. These findings establish optimized protocols and identify potential genomic determinants of transformability under the rapid agar-lawn protocol, broadening genetic accessibility for functional genomics and pathogenesis studies in *L. monocytogenes*.

## Highlights

• 32 mM cyclic AMP supplementation enhances transformation efficiency by up to 377-fold.
• cAMP enhancement is transient, efficiency peaks immediately postsupplementation.
• The rapid agar–lawn protocol cuts electrocompetent cell preparation from ten hours to under three hours.
• Eighty–three percent of 66 *L. monocytogenes* isolates were transformable using the rapid agar–lawn protocol.
• Defence–antidefence network analysis links immune system repertoires to transformability.

*L. monocytogenes* is a Gram-positive, facultative intracellular bacterium and a prominent foodborne pathogen responsible for severe illness, including meningitis, septicemia, and fetal loss, particularly in immunocompromised individuals (Authority (EFSA) & European Centre for Disease Prevention and Control (ECDC), 2023, Radoshevich and Cossart, 2018). *L. monocytogenes* is structured into four major phylogenetic lineages (I–IV), encompassing more than 100 defined clonal complexes (CCs), each associated with characteristic ecological and epidemiological patterns (Moura et al., 2016). Lineage I CCs, such as CC1 and CC2, are strongly linked to human listeriosis and hypervirulence, whereas lineage II CCs, including CC7, CC9, and CC121, are frequently isolated from food and food-processing environments and display enhanced stress-tolerance traits (Maury et al., 2016, Moura et al., 2016, Ragon et al., 2008). Beyond its public health significance, *L. monocytogenes* has emerged as a model organism to study host-pathogen interactions, intracellular survival, and immune evasion (Freitag et al., 2009, Pizarro-Cerdá and Cossart, 2018).

Elucidating the molecular basis of *L. monocytogenes* virulence and physiology relies heavily on genetic manipulation. However, most studies have been conducted using a limited number of laboratory-adapted reference strains, such as EGD-e, 10403S, and EGD, which do not reflect the genomic, ecological, or phenotypic diversity observed across clinical and environmental isolates (Bécavin et al., 2014, Maury et al., 2016). Recent population-level studies have shown substantial variation in stress resistance, virulence potential, and phage sensitivity among *L. monocytogenes* lineages, raising concerns about the generalizability of findings based on laboratory-adapted strains (Disson et al., 2021, Kawacka et al., 2020, Lakicevic et al., 2022, Mao et al., 2023, Muchaamba et al., 2022).

Several tools for genetic manipulation in *L. monocytogenes,* such as allelic exchange vectors, shuttle plasmids, and CRISPR interference (CRISPRi) systems, are available (Argov et al., 2017, Hupfeld et al., 2018, Monk et al., 2008, Peters et al., 2019). For example, a novel suicide vector incorporating a *pheS* (phenylalanyl-tRNA synthetase) counterselection system enables efficient, markerless allelic exchange in *L. monocytogenes* (Argov et al., 2017). Additionally, a series of phage integrase–based vectors (pIMK, pIMK2/3/4) has been developed for chromosomal integration and gene overexpression, along with an optimized replicative plasmid system (pORI280) for generating chromosomal deletions (Monk et al., 2008). In the same study, Monk et al. significantly improved electroporation protocols, establishing a gold standard method. They systematically adjusted every step of the workflow, including mid‐log phase growth in sucrose-supplemented Brain Heart Infusion (BHI) to ensure active division and osmotic protection, ampicillin pretreatment to slow peptidoglycan synthesis and loosen the cell wall, lysozyme digestion to partially degrade peptidoglycan, multiple cold sucrose‐glycerol washes to remove conductive ions and preserve viability, and final electroporation at 10 kV/cm (25 µF, 400 Ω). Under these conditions, transformation efficiencies differed between strains. While EGDe and 10403S reached approximately 10⁶ CFU/µg DNA using 10 µg/mL lysozyme pretreatment, strain F2365 achieved approximately half a log higher efficiency when treated with 25 µg/mL lysozyme. Conversely, applying 25 µg/mL lysozyme to EGDe and 10403S, or 10 µg/mL to F2365, resulted in an approximate 50% reduction in transformation efficiency (Monk et al., 2008), illustrating that transformation output is strain dependent and influenced by cell wall weakening. In contrast, model organisms like *Escherichia coli* and *Bacillus subtilis* possess robust, high-efficiency transformation systems, including inducible natural competence and streamlined recombineering platforms (Calero and Nikel, 2019, Put et al., 2024, Stülke et al., 2023, Wu et al., 2019). Natural competence, a physiological state in which bacteria uptake exogenous DNA, is regulated in many species by environmental cues, like nutrient limitation (Blokesch, 2016, Dubnau and Blokesch, 2019, Seitz and Blokesch, 2013), often mediated by cAMP and its receptor protein, cAMP receptor protein (CRP) (Cameron and Redfield, 2008, Findlay Black et al., 2020, Hu et al., 2022, Wu et al., 2015). For instance, in *Haemophilus influenzae* and *Vibrio cholerae*, cAMP-CRP signaling activates competence genes and facilitates horizontal gene transfer (Blokesch, 2012, Dorocicz et al., 1993). *L. monocytogenes*, however, despite carrying genes associated with natural competency, lacks a functional natural competence system and does not naturally acquire DNA under standard laboratory conditions (Borezee et al., 2000, Goh et al., 2024, Johansson and Freitag, 2019, Rabinovich et al., 2012).

Other defense barriers against foreign DNA could impact transformation outcomes, such as restriction-modification (RM) systems, CRISPR-Cas machinery, and abortive infection (Abi) systems (Stern and Sorek, 2011, Xu and Gu, 2024). There is a potential for variability in transformability, as for example, some *L. monocytogenes* isolates harbor multiple active RM systems or MGEs encoding defense systems (Benler and Koonin, 2022, Brown et al., 2023, Gutiérrez et al., 2025).

We aimed to optimize and broaden transformation protocols for diverse *L. monocytogenes* isolates. First, we tested whether increasing electroporation voltage and supplementation with cAMP could enhance DNA uptake across genetically distinct isolates. Next, we developed a rapid agar‐lawn method to streamline the transformation workflow. Finally, we screened 66 clinical, environmental, and food-related isolates to examine the relationship between transformation efficiency and genomic features, such as plasmid and prophage content, as well as defense/antidefense repertoires, using co‐occurrence network analysis. This integrated approach aimed to improve accessibility to transformation and identify common genetic features among nontransformable isolates.

## Materials and methods

### Bacterial isolates and plasmid preparation

A total of 66 *L. monocytogenes* isolates (Supplementary Table 1) were selected from an internal collection maintained at the Quadram Institute Bioscience, originally assembled through i) research activities led by Dr. Barbara Lund, including shared isolates and retail sampling conducted at the former Institute of Food Research, and ii) collaborative agreements with UK Health Security Agency (UKHSA) and the Public Health Agency of Canada. Isolates were selected to represent diverse isolation sources and major genetic lineages.

Plasmids pHPL3-mCherry (Mitchell et al., 2018), pBAV1-T5-GFP (Bryksin & Matsumura, 2010), pKSV7 (Smith & Youngman, 1992), and pJZ037 (Zemansky et al., 2009) (Table 1), were propagated and extracted from High Efficiency *E. coli* DH5α (New England Biolabs; Ipswich, MA, USA) using the commercially available HiSpeed Maxi kit (Qiagen; Hilden, Germany), following the manufacturer's protocol, with plasmid elution performed in Ultrapure water (Sigma Aldrich; St. Louis, MO, USA). Plasmid extracts with concentrations below 100 ng/μL underwent ethanol precipitation to increase DNA yield. Briefly, 0.1 vol of 3 M sodium acetate (Sigma Aldrich), three volumes of ice-cold 100% ethanol (VWR Chemicals; Radnor, PA, USA) were added to the plasmid extract and vortexed thoroughly. The mixture was stored at −80 °C for 1 h, then centrifuged at 12,000 g, 4 °C for 30 min (5800R Centrifuge, Eppendorf; Hamburg, Germany). The resulting pellet was washed twice with 0.5 mL ice-cold 75% ethanol, centrifuging at 13,000 rpm and 4 °C for 10 min each time. After removing the ethanol, the pellet was air-dried and resuspended in Ultrapure water (Sigma Aldrich).

**Table 1:** Plasmids used in this study

Plasmid	Replication mode	Size	Selection	Reference
pHPL3-mCherry	Integrative	7.7 Kbp	Chloramphenicol 7.5 μg/ml	(Mitchell et al., 2018)
pBAV1-T5-GFP	Replicative	3.6 Kbp	Kanamycin 50 μg/ml	(Bryksin & Matsumura, 2010)
pKSV7	Replicative	7 Kbp	Chloramphenicol 7.5 μg/ml	(Smith & Youngman, 1992)
pJZ037	Replicative	9.2 Kbp	Chloramphenicol 7.5 μg/ml	(Zemansky et al., 2009)

For antibiotic selection, the following concentrations were used: chloramphenicol, 7.5 μg/mL and kanamycin, 50 μg/mL (Sigma Aldrich) (Table 1)*.*

Adenosine 3′,5′-cyclic monophosphate sodium salt monohydrate (cAMP) was purchased from Sigma Aldrich, prepared as a working solution of 500 mM (184.6 mg of cAMP in 1 mL of Ultrapure water), and stored at −20 °C in aliquots.

### Gold standard *Listeria* electrocompetent preparation (Monk et al., 2008) with modifications

Bacterial isolates were cultured overnight in 20 mL of BHI (Oxoid; Hampshire, UK) at 37 °C with 180 rpm shaking (New Brunswick™ Innova® 42 Incubator Shaker; Edison, NJ, USA). Overnight precultures were used to inoculate 500 mL of BHI supplemented with 500 mM sucrose (Fisher Chemicals, Waltham, MA, USA) to an initial optical density (OD_600_) of 0.01–0.02 and incubated at 37 °C with shaking (180 rpm) until mid-log phase, approximately three hours, reaching an OD_600_ of 0.2–0.22 (not exceeding 0.25). At this point, a final concentration of 10 μg/mL ampicillin (Sigma Aldrich) was added, and the culture was incubated for two additional hours at 37 °C with shaking (180 rpm). The cells were then placed on ice for 10 min and pelleted by centrifugation (Eppendorf® 5800R Centrifuge) at 3,500 g for 10 min at 4 °C. The bacterial pellet was resuspended in 250 mL of cold wash buffer (10% glycerol and 500 mM sucrose, adjusted to pH 7.0), sterilized by filtration using a LABOPORT pump (KNF Neuberger; Freiburg-Munzingen, Germany) and a 0.22 μm filter (Starlab; Houston, TX, USA), and centrifuged again at 3,500 g for 20 min at 4 °C. This wash step was repeated twice, with resuspension of 150 mL and 50 mL, respectively. Subsequently, 50 μL of lysozyme (Sigma Aldrich, 10 mg/mL in PBS, 0.22 μm filter-sterilized [Fisher Scientific UK Ltd, Loughborough, Leicestershire, UK]) was added to the suspension to achieve a final concentration of 10 μg/mL, and the mixture was incubated for 20 min at 37 °C statically. For experiments requiring a final concentration of 25 μg/mL of lysozyme, 125 μL of the solution was added to the 50 mL bacterial resuspension. The cells were then pelleted by centrifugation (Eppendorf® 5800R Centrifuge) at 2,500 g for 20 min and resuspended in 20 mL wash buffer. A final centrifugation (Eppendorf® 5800R Centrifuge) was performed, and the pellet was resuspended in 2.5 mL of wash buffer. Aliquots of 50 μL of the electrocompetent cells were stored at −80 °C.

### Rapid agar-lawn *Listeria* electrocompetent preparation

Bacterial isolates were cultured overnight in BHI at 37 °C with shaking (180 rpm). To generate a bacterial lawn, 100–200 μL of the overnight culture was spread evenly onto two BHI agar plates (100 mm) (Oxoid), which were then incubated at 37 °C for 24–96 h. After incubation, the bacterial biomass was harvested by gently scraping the agar with a sterile loop (Thermo Fisher Scientific; Waltham, MA, USA) and resuspended in 50 mL of ice-cold PBS.

As a control condition, electrocompetent cells were also prepared from bacteria grown exclusively in broth suspension (hereafter referred to as planktonic cultures). For this, BHI broth was inoculated and incubated overnight (approximately 16 h) at 37 °C with shaking (180 rpm). This comparison enabled assessment of whether growth on solid medium (agar lawn) influenced competence relative to planktonic growth prior to electrocompetent cell preparation.

Cells harvested from lawns or grown planktonically were pelleted (Eppendorf® 5800R Centrifuge) at 3,500 g for 10 min at 4 °C. The supernatant was carefully discarded to avoid disturbing the pellet, and the cells were gently resuspended in 50 mL of ice-cold wash buffer. This washing step was repeated under the same conditions, and the resulting pellet was resuspended in 500 μL of wash buffer and divided into 50 μL aliquots for storage at −80 °C.

### Transformation of electrocompetent *Listeria*

In a chilled 2 mm electroporation cuvette (Mirus Bio LLC; Madison, WI, USA), 2 μg of plasmid DNA was combined with 50 μL of electrocompetent bacteria and gently mixed. Where indicated, cAMP, ranging from 4 to 64 mM final concentration, was added just before electroporation. Electroporation using a Gene Pulser® II Electroporation System (Bio-Rad; Hercules, CA, USA) was performed immediately using the following settings: 400 Ω, 2.2 KV (11 Kv/cm) or 2 KV (10 Kv/cm), and 25 μF. The cuvette immediately returned to ice following pulse delivery. To allow cell recovery, 300 μL of BHI supplemented with 500 mM sucrose was added to the cuvette and incubated at 37 °C statically for 1.5 h. When working with the pJZ037 plasmid, the suspension was incubated at 30 °C statically for 1.5 h instead. After recovery, the cells were plated on BHI agar containing the appropriate antibiotic (Table 1) and incubated at 37 °C, or at 30 °C for pJZ037. Visible colonies typically appeared within 24 to 48 h.

Screening of 66 electrocompetent isolates using the rapid agar-lawn transformation protocol was performed by combining 50 μL of electrocompetent cells with 2 μg of plasmid pHPL3-mCherry and 32 mM cAMP, followed by gentle mixing and immediate electroporation (400 Ω, 2.2 KV (11 Kv/cm), and 25 μF). Recovery and plating steps were carried out as described earlier.

### DNA extraction and sequencing

DNA was extracted from 57 bacterial isolates (this study, see genome assemblies section for details), following two different methods: DNA used for short-read sequencing was extracted from 800 µL of overnight culture in BHI using the automated Maxwell® RSC 48 instrument and the Maxwell® RSC Cultured Cells DNA (Promega; Madison, WI, USA) kit following the manufacturer’s instructions; DNA used for long-read sequencing was purified from 1 mL of overnight culture in BHI using the Fire Monkey High Molecular Weight DNA (HMW-DNA) extraction kit (RevoluGen Ltd; Berkshire, UK) after treating the cells with 20 mg/mL lysozyme (Roche®; Basel, Switzerland) and 1.2% Triton X-100 (Sigma Aldrich) for 30 min at 37 °C and 180 rpm shaking. DNA was quantified using the QuantiFluor® dsDNA System and GloMax® Discover Microplate Reader (Promega). Paired-end short read sequencing libraries were prepared with the Illumina DNA Prep kit, and 150 bp paired-end reads were sequenced using the Illumina Nextseq500 instrument with a NextSeq 500/550 Mid Output Kit v2 (300 Cycles) (Illumina, Inc.; San Diego, CA, USA). Long-read sequencing libraries were prepared using the nanopore ligation sequencing kit (SQK-LSK109) in combination with the native barcodes (EXP-NBD196) and sequenced using a SpotON flow cell (R9.4.1) and a MinION sequencing device. Data acquisition was performed using MinKNOW v3.1.8 software. Base calling and adapter trimming were done using the high-accuracy mode of the basecaller Guppy v5.0.12+eb1a981 (Oxford Nanopore Technologies, Oxford, UK).

### Bioinformatic analysis

**Genome assemblies.** Hybrid assemblies (closed genomes) of 55 isolates from Quadram Institute Bioscience and UKHSA collections (Bioproject PRJNA248549; Supplementary Table 1) were generated as previously described (Gutiérrez et al., 2025) and are available through the National Center for Biotechnology Information (NCBI) under BioProject PRJNA837734. Reads for isolates BL86-016 and BL87-009 are also available at BioProject PRJNA837734. Draft assemblies of these two isolates were constructed using shovill v1.1.0 (Seemann, 2016/2024) with SPAdes v3.14 selected as assembler (Bankevich et al., 2012) from quality−trimmed reads generated using TrimGalore v0.4.3 (Krueger et al., 2023) and are publicly available at https://doi.org/10.5281/zenodo.18390987. Genome assemblies of the remaining nine isolates from the Public Health Agency of Canada were accessed through NCBI under Bioprojects PRJNA36361, PRJNA167893, PRJNA167895, PRJNA167875, PRJNA167878, PRJNA167882, PRJNA167880, PRJNA167881, and PRJNA215191. The authors confirm that all supporting data and protocols have been provided within the article or through supplementary data files.

For clonal complex and lineage classification, genomes were typed using MLST v2.16.1 (Seemann, 2014) with the PubMLST database (Jolley & Maiden, 2010).

Identification of single-nucleotide polymorphism (SNP) differences between the reference strains F2365 (GCF_000008285.1) and EGD-e (GCF_000196035.1) against BL87-016 and BL91-025, respectively, was identified using Snippy v3.2 (Seemann, 2015) using default parameters.

**BLAST analyses.** Plasmids were identified by MOB-suite v3.0.0 under default settings (Robertson & Nash, 2018). Plasmids derived from 18 out of 66 isolates were aligned to the NCBI nucleotide collection database using the web-based Basic Local Alignment Search Tool (BLAST) under default settings (Altschul et al., 1990, Johnson et al., 2008). GenBank accessions of hits with >99% identity were parsed against the replication system groups identified by Chmielowska et al. (2021).

**Defense‐systems detection.** Defense and antidefense systems were detected using the web tool DefenseFinder v2.0.0 under default parameters (Tesson et al., 2024).

**Prophage and *comK* disruption detection.** Prophages were identified from genome assemblies (hybrid and draft assemblies) using Vibrant v1.2.1. (Kieft et al., 2020).

To assess *comK* disruption, the assemblies of the 66 genomes were first annotated using Bakta v1.6.1 (Schwengers et al., 2021), which are publicly available at https://doi.org/10.5281/zenodo.18390987. The full-length *comK* gene (609 bp) (Kwon et al., 2020) was manually identified in the annotated assemblies, and the absence of prophage within the *comK* gene was cross-verified with the results from prophage detection.

**Phylogenetic reconstruction.** The phylogeny of 66 isolates was constructed as described previously (Gutiérrez et al., 2025). Briefly, assemblies were aligned to the reference genome EGD-e (GCF_000196035.1) using Snippy v3.2 (Seemann, 2015). The reference genome was masked for repeat regions using BEDTools v2.29.0 (Quinlan, 2014), and a minimum base quality of 20, read coverage of 10X, and 90% read concordance were used as criteria for variant calling. Monomorphic and variant sites were used to determine the total core genome size, which was computed using snp-sites v2.5.1 (Page et al., 2016). Gubbins v2.4.1 (Croucher et al., 2015) was employed to detect potential recombination regions, and any identified regions were masked from the sequences. The recombination-purged variant sites were obtained by only including sites with A, C, G, or T using snp-sites v2.5.1 (Page et al., 2016). RaxML v8.2.4 (Stamatakis, 2014) was used to generate maximum likelihood phylogenetic trees using the sequence alignment for these sites, employing the GTRCAT model. Finally, IQ-TREE v2.2.6 (Minh et al., 2020, Nguyen et al., 2015) was used to correct for ascertainment bias using the −fconst flag with both the full alignment and “N” sites as input, employing the HKY+G model. The phylogenetic reconstruction was mid-rooted in R (v2022.02.2) using phytools v1.0-3 (Revell, 2012).

**Construction of defense and antidefense system cooccurrence networks.** All data parsing and analysis were performed in Python (v3.10) using pandas (v1.5.3), NetworkX (v2.8.8), and Matplotlib (v3.6.2). Cooccurrence networks were inferred using a presence/absence matrix of defense and antidefense systems across isolates (Faust and Raes, 2012, Lima-Mendez et al., 2015). Isolates were classified as either transformable or nontransformable based on experimental assessments of competence using the rapid agar-lawn method described in this study. For each group, a symmetric cooccurrence matrix was computed as $C=P^{T}P$, where C is the symmetric cooccurrence matrix, P is the binary presence/absence matrix of defense systems (with isolates in rows and systems in columns), and $P^{T}$ is the transpose of P. Each element $C_{\mathrm{ab}}$​ represents the number of isolates carrying both defense systems a and b. Diagonal entries (self-cooccurrence) $C_{\mathrm{aa}}$​ were set to zero to focus strictly on pairwise system associations. Cooccurrence networks were then built by retaining only edges with a cooccurrence count of two or greater (number of isolates in which both systems cooccur), ensuring that only robust and repeatedly observed interactions were visualized. Network layouts were generated using a force-directed Fruchterman–Reingold algorithm (Fruchterman & Reingold, 1991), and node sizes were scaled in proportion to degree centrality. To quantify and compare topological features between transformable and nontransformable networks, we computed network density, average clustering coefficient, and modularity (via the fast-greedy community detection algorithm) in NetworkX (Brandes et al., 2008, Newman and Girvan, 2004).

### Statistical analysis

All experimental procedures were performed with three biological replicates. Results are presented as mean values with error bars representing the standard deviation, using GraphPad Prism v5.04 for statistical analysis and plotting. Statistical significance between two groups was assessed using a *t*-test. Comparisons between a control group and multiple treatment groups were performed using one-way ANOVA with Dunnett's posthoc test, while comparisons among multiple groups were assessed using one-way ANOVA with posthoc Tukey’s test. Nonsignificant differences are denoted as ‘NS’ (*p* > 0.05), and significant differences as * (*p* ≤ 0.05), ** (*p* ≤ 0.01), and *** (*p* ≤ 0.001).

To assess whether transformability under the rapid agar-lawn protocol was related to lineage or MGEs, we performed statistical tests tailored to the data structure of each categorical variable using Pearson’s chi-square test of independence. All analyses were performed in R v2022.02.2, with a *p* < 0.05 considered significant. Transformability was treated as a binary outcome. Lineage-specific associations were evaluated for Lineages I and II, while lineage III was excluded from lineage-specific pairwise comparisons due to insufficient sample size (<3) for reliable inference. Plasmid carriage was analyzed both as a binary variable (presence vs. absence) and as a three-level categorical variable capturing predicted mobility class (conjugative, mobilizable, or nonmobilizable). Although considered, associations involving replication proteins (repA groups G1 vs. G2) were not evaluated due to small counts of strains carrying them.

Quantitative genomic features were compared between transformable and nontransformable isolates using Welch’s *t*-test, which accounts for unequal variances and group sizes and a nonparametric Mann–Whitney *U* test performed as a confirmatory analysis to ensure robustness to deviations from normality. The cumulative burden of defense and antidefense systems was quantified for each genome by summing the total number of detected defense and antidefense loci per isolate. The number of intact prophages per genome and total defense-system counts were analyzed using this approach.

The integrity of the competence regulator *comK* (intact vs. disrupted) was treated as a binary categorical variable. Associations between *comK* status and transformability were tested using Fisher’s exact test, given the small number of disrupted alleles in the nontransformable group.

## Results and discussion

### Increase in voltage optimizes transformation of *L. monocytogenes*

Transformation efficiency, calculated as the number of transformants per microgram of DNA, is a key parameter in assessing how effectively exogenous DNA can be introduced into bacterial cells (Park & Stewart, 1990). In *L. monocytogenes*, published methods focus on reference laboratory strains (e.g., F2365, EGDe), which routinely achieve efficiencies of ∼10^6^–10^7^ CFU/μg DNA (Monk et al., 2008). However, isolates sourced from food, environment, and clinical samples often transform poorly or not at all. Among closely related isolates, differences in transformability have been reported in several bacterial species (Gaasbeek et al., 2009, Mazzamurro et al., 2024, Shambhu et al., 2024), but no such comparisons exist for *L. monocytogenes*. This gap highlights the need to refine current “gold standard” transformation protocols for broader applicability.

To develop a transformation protocol with broader performance, we evaluated whether modifications (increase in voltage and lysozyme concentration) to the current gold standard method for preparing electrocompetent cells (Monk et al., 2008, Rychli et al., 2014) could enhance transformation across diverse *L. monocytogenes* genetic backgrounds. We selected three isolates representing both closely related to widely used laboratory reference strains and epidemiologically relevant clonal complexes: BL87-016 (CC1; 39 SNPs relative to reference strain F2365), BL91-025/ATCC 19111 (CC7; 27 SNPs relative to reference strain EGD-e), and BL87-028-B [CC121; a chicken isolate lacking the prophage integrated in *comK*, derived from the published BL87-028 isolate (Gutiérrez et al., 2025)] (Supplementary Table 1). BL87-016 and BL91-025 isolates are therefore closely related representatives of the widely used reference strains F2365 and EGD-e, respectively, allowing us to assess whether the enhanced protocol improves performance in canonical laboratory genetic backgrounds. In contrast, CC121 is one of the most prevalent clonal complexes in food and food-processing environments and is frequently associated with environmental persistence (Knudsen et al., 2017). Its inclusion, therefore, provides an important test of protocol performance in a nonlaboratory-adapted background. Additionally, four plasmids were included for testing transformation efficiency: one integrative (pPL3-mCherry) and three replicative (pBAV1-T5-GFP, pKSV7, pJZ037) plasmids reflecting different replicons (Table 1).

**Figure 1:**
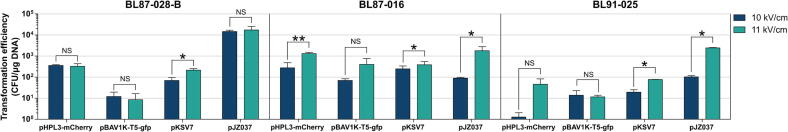
**Voltage-dependent enhancement of transformation efficiency across *L. monocytogenes* isolates and plasmids.** Transformation efficiency (CFU/μg DNA) was measured for three *L. monocytogenes* isolates (BL87-016, BL91-025, and BL87-028-B) using four plasmids: the integrative pPL3-mCherry and the replicative pJZ037, pBAV1-T5-GFP, and pKSV7. Electroporation was performed at standard (10 kV/cm) and elevated (11 kV/cm) voltages to assess improvements in DNA uptake. Data represent mean transformation efficiencies ± SD from at least three independent experiments. Statistical significance was determined using two-tailed Student’s t tests. Nonsignificant differences are denoted as NS (*p* > 0.05), and significant differences as * (*p* ≤ 0.05), and ** (*p* ≤ 0.01).

Increasing the electroporation voltage from the standard 10 kV/cm to 11 kV/cm in the gold standard protocol yielded improvements in transformation efficiency for about half of the isolate–plasmid combinations and did not reduce the efficiency in any isolate-plasmid combination. For the replicative pJZ037, transformation increased nearly 20-fold in BL87-016 and 24-fold in BL91-025; the integrative pPL3-mCherry showed 4.7- and 36-fold improvements in the same isolates, respectively (Fig. 1). In contrast, BL87-028-B exhibited no differences when transformed with pJZ037 and pPL3-mCherry. Intermediate increases were observed with pKSV7 (1.6- to fourfold across isolates) and pBAV1-T5-GFP (nearly sixfold in BL87-016 but unchanged or slightly reduced in the other isolates) (Fig. 1).

Although Monk et al. (Monk et al., 2008) reported that for the gold standard protocol, increasing lysozyme concentration from 10 μg/mL to 25 μg/mL improved transformation efficiency in strain F2365 by approximately twofold, our study found the opposite effect in the closely related isolate BL87-016. Specifically, transformation efficiency was reduced by 0.4–0.9-fold (not statistically significant) when using 25 μg/mL lysozyme compared to 10 μg/mL (Supplementary Fig. 1), indicating that excessive enzymatic degradation of peptidoglycan might compromise cell integrity during transformation protocols. The reduction of *L. monocytogenes* viability by lysozyme has been previously described, where 35 μg/mL lysozyme reduced cell viability from 5.1 log CFU/mL to below the detection limit within 3 h (Yang et al., 2007). Taken together, our data indicate that the optimal balance for *L. monocytogenes* transformation is 10 μg/mL lysozyme pretreatment coupled with 11 kV/cm electroporation. Under these conditions, we achieved up to 1.74 × 10^4^ CFU/μg DNA with pJZ037 in isolate BL87-028-B (Fig. 1), maximizing DNA uptake while preserving viability.

### Use of *cAMP* enhances transformation of *L. monocytogenes*

Although cAMP enhances natural competence in several bacterial species through CRP-mediated regulation (Bigas et al., 2005, Blokesch, 2012, Wang et al., 2002, Wise et al., 1973), this effect is highly species-specific (Bossé et al., 2004, Guo et al., 2015). To our knowledge, in *L. monocytogenes*, natural competence has not been reported, and despite our extensive efforts, including repeated attempts using cAMP supplementation and different growth conditions, no transformants were recovered.

**Figure 2:**
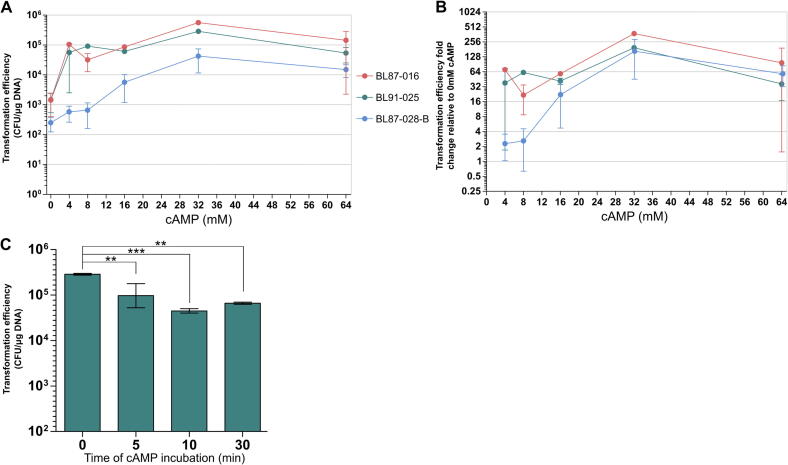
**Camp enhances transformation efficiency in *L. monocytogenes* in a dose- and time-dependent manner.** (A) Transformation efficiencies (CFU/µg DNA) and (B) transformation efficiency fold change for three *L. monocytogenes* isolates, BL87-028-B, BL87-016, and BL91-025, were evaluated across increasing concentrations of cAMP (0–64 mM) added immediately before electroporation. (C) Time-course analysis in BL91-025 demonstrated that the transformation-enhancing effect of 32 mM cAMP is transient. Data represent the mean ± standard deviation of at least three independent experiments. Statistical significance in panel C was assessed using one-way ANOVA with posthoc Dunnett’s test. Significant differences are denoted as ** (*p* ≤ 0.01) and *** (*p* ≤ 0.001).

Given this, we explored whether cAMP could instead enhance *L. monocytogenes* transformation efficiency when introduced during electroporation. A dose-dependent improvement in transformation efficiency across three genetically distinct isolates occurred when cAMP was added prior to electroporation (Fig. 2A–B). The greatest effect was observed at 32 mM cAMP. In BL87-028-B, the transformation efficiency rose from 2.48 × 10^2^ CFU/µg DNA to 4.12 × 10^4^ CFU/µg DNA (Fig. 2A). BL87-016 exhibited a jump from 1.42 × 10^3^ to 5.37 × 10^5^ CFU/µg DNA, while BL91-025 increased from 1.47 × 10^3^ to 2.87 × 10^5^ CFU/µg DNA (Fig. 2A).

To our knowledge, this is the first demonstration that cAMP through transformation enhances competence. While cAMP-mediated enhancement of natural competence is well documented, often resulting in 10- to 1000-fold increases depending on species and conditions (Bigas et al., 2005, Wang et al., 2002), its use in artificial transformation protocols such as electroporation has not been previously reported. To further understand the kinetics of this phenomenon, we focused on isolate BL91-025 as a representative intermediate responder (∼195-fold increase at 32 mM cAMP), compared with BL87-028-B (∼166-fold) and the higher-responding isolate BL87-016 (∼377-fold) (Fig. 2B). The concentration of 32 mM cAMP was selected for subsequent experiments as it consistently produced maximal transformation efficiency across all three isolates (Fig. 2A). BL91-025 was therefore used to assess the impact of incubation time following cAMP addition. Electroporation performed immediately after adding 32 mM cAMP (“0 min”) yielded the peak transformation efficiency of 2.87 × 10^5^ CFU/µg DNA (Fig. 2C). A five-minute stagger from cAMP exposure to transformation reduced efficiency to ∼1.08 × 10^5^ CFU/µg DNA, and a ten-minute delay reduced efficiency further to ∼4.5 × 10^4^ CFU/µg DNA. Notably, a 30-minute stagger produced a partial rebound in efficiency (∼6.62 × 10^4^ CFU/µg DNA), though still below the initial maximum observed (Fig. 2C). We believe that the competence-enhancing effect of cAMP is not only dose-dependent but also highly transient, likely reflecting a short-lived physiological state or rapidly reversible membrane modulation.

### Balancing efficiency and practicality: A rapid agar-lawn method for *L. monocytogenes* transformation

**Figure 3:**
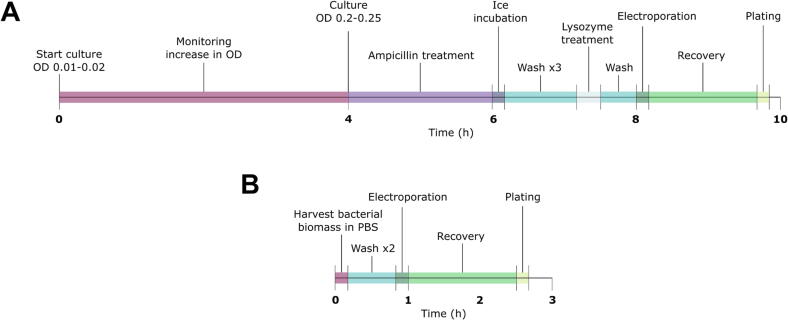
Comparison of workflow timelines for electrocompetent *L. monocytogenes* preparation. (A) Schematic representation of the gold standard protocol (Monk et al., 2008). (B) Streamlined protocol was developed in this study, using bacterial lawns grown on BHI agar. The simplified workflow is compatible with cAMP supplementation and electroporation, offering a faster alternative for routine applications. See more details in the Materials and Methods section.

Genetic manipulation of *L. monocytogenes* presents a technical challenge that requires balancing transformation efficiency with time and workload. The commonly used and gold standard transformation protocol developed by Monk et al. (Monk et al., 2008, Rychli et al., 2014) has remained the most reliable method for high-efficiency transformation in this species. Our optimizations, increasing the electroporation voltage and cAMP supplementation, resulted in 100–300-fold improvements in competence compared to the original gold standard methodology (Fig. 2AB). The original and optimized gold standard protocols require a significant time commitment, approximately 10 h from initial culture to plating of transformed cells (Fig. 3A).

To address this limitation, we developed a simplified transformation workflow that replaces time-intensive liquid culture preparation with a solid-medium approach. In this method, *L. monocytogenes* is grown as a lawn on BHI agar, harvested, followed by only two washes, and then subjected directly to cAMP treatment and electroporation. By eliminating lysozyme treatment, reducing the number of total washing steps, and bypassing extended culture periods, hands-on time is reduced to under three hours (Fig. 3B). Because lawn-grown cells likely exist in a stationary state, we also prepared electrocompetent cells from planktonic cultures harvested at the stationary phase for comparison. Rapid electrocompetent cells were generated from lawns incubated for 24 to 96 h, as well as from overnight BHI broth cultures.

**Figure 4:**
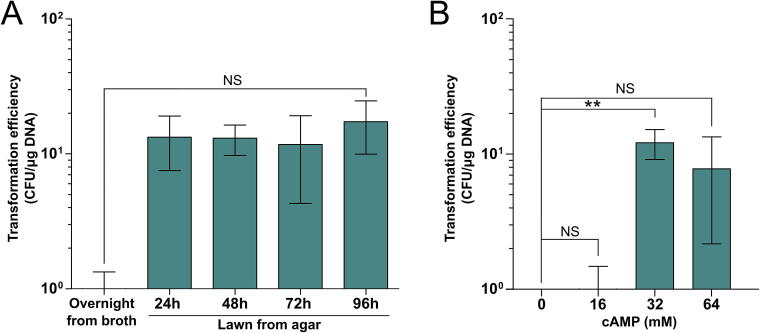
**Evaluation of transformation efficiencies using the streamlined agar-lawn protocol.** (A) Transformation efficiencies (CFU/µg DNA) for *L. monocytogenes* BL87-028-B using electrocompetent cells derived from overnight liquid culture or 24–96 h BHI agar lawns. Statistical significance was assessed using one-way ANOVA with posthoc Tukey’s test. (B) Effect of cAMP supplementation on transformation efficiency using agar-grown cells. Statistical significance was assessed using one-way ANOVA with posthoc Dunnett’s test. Data represent the mean ± standard deviation of at least three independent experiments. Nonsignificant differences are denoted as NS (*p* > 0.05), and significant differences as ** (*p* ≤ 0.01).

While simplifying the experimental workflow, this streamlined protocol yielded markedly lower transformation efficiencies (3–5 log) than the optimized gold standard protocol (Supplementary Fig. 2). Cells harvested from overnight liquid cultures yielded fewer than five transformants per microgram of DNA. In contrast, agar-grown cells had modestly higher efficiencies: 24-hour lawns produced an average of 13.3 CFU/µg DNA, which increased slightly to approximately 17 CFU/µg by 48–96 h (Fig. 4A). These results are orders of magnitude lower than the optimized gold standard protocol (Supplementary Fig. 2), yet they present practical benefits in select experimental contexts. Notably, as with the optimized gold standard protocol, electrocompetent cells derived from 48 h of agar lawns exhibited maximum transformation efficiency at 32 µM cAMP, while without cAMP, no transformants were observed (Fig. 4B).

The optimized gold standard protocol remains the method of choice for applications that require large numbers of transformants, such as transposon mutagenesis screens, creation of CRISPRi libraries, or introduction of low-copy or large plasmids. However, the rapid agar-lawn protocol offers a valuable alternative for routine transformations where time, simplicity, and resource efficiency are prioritized over maximum yield. Specifically, this includes routine plasmid expression studies, diagnostic cloning, or other applications in which transformation efficiency is not the limiting factor. Previous work has shown that electrocompetent *L. monocytogenes* prepared using the gold standard protocol can be stored at −80 °C for at least six months without loss of transformability (Monk et al., 2008). While rapid agar-lawn–derived electrocompetent cells are suspended in the same sucrose–glycerol buffer, their long-term storage stability was not assessed here and remains to be determined in future studies.

### Genomic determinants of competence in *L. monocytogenes* reveal diverse defense and antidefense network architectures

To assess our rapid agar-lawn transformation protocol using a broader spectrum of ecological and genetic backgrounds of *L. monocytogenes*, we screened 66 isolates sourced from clinical, environmental, and food-processing environments. Our primary aim was to evaluate whether this streamlined approach could consistently generate electrocompetent cells across all major phylogenetic lineages (I-III) and where sample size permitted statistical testing, to determine whether competence was associated with specific genomic features.

**Figure 5:**
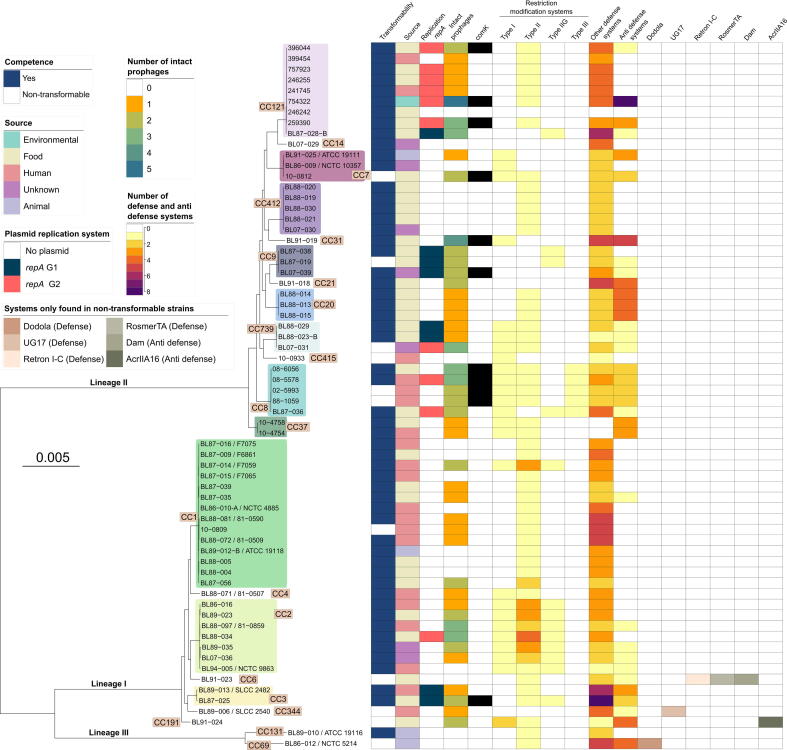
**Phylogenetic distribution of transformation efficiency and genomic features across 66 *L. monocytogenes* isolates.** A midpoint-rooted maximum likelihood tree, corrected for ascertainment bias, was constructed based on a recombination-purged SNP core alignment of 66 *L. monocytogenes* isolates, using EGD-e (GCF_000196035.1) as the reference genome. Clonal complexes represented by two or more isolates are marked in distinct colors. Associated metadata, including transformation outcome, plasmid carriage, prophage content, *comK* disruption status, and the presence of selected defense and antidefense systems, are overlaid. Black boxes denote disruption of the *comK* gene. The scale bar represents branch length measured in substitutions per site.

The rapid agar-lawn transformation protocol proved broadly effective, with competence observed for 83% of isolates (55 out of 66; Fig. 5). For the purposes of this study, isolates that yielded at least one antibiotic-resistant, red fluorescent colony following electroporation were classified as “transformable”, whereas isolates that produced no colonies under the same conditions were classified as “nontransformable”. Although 11 isolates did not transform under the rapid agar-lawn protocol, we cannot exclude the possibility that some of these isolates might transform under more intensive conditions. We integrated key metadata, such as competence, plasmid carriage, prophage content, and the presence of bacterial defense and antidefense systems, into a maximum‐likelihood phylogeny constructed from core‐genome alignments (Fig. 5). Consistent with earlier observations (Mazzamurro et al., 2024, Shambhu et al., 2024), our dataset also revealed transformation variability within clonal complexes. For example, while isolates BL87-016 (CC1) and BL91-025 (CC7) were transformable, other members of the same clonal complexes (e.g., 10-0809, 10-0812, respectively) were not, despite their genetic relatedness (Fig. 5).

A chi-square test of independence was performed to examine the relationship between transformability and phylogenetic lineages, which was not significant (χ^2^ = 0.023, *p* = 0.88), as 85% of lineage I isolates and 84% of lineage II isolates were classified as transformable, with the remaining isolates in each lineage being nontransformable. Similarly, transformation capacity did not correlate with plasmid carriage (χ^2^ = 0.550, *p* = 0.46) or mobility class (χ^2^ = 3.99, *p* = 0.26): 29.1% of transformable isolates carried plasmids compared to 18.2% of nontransformable isolates (Fig. 5).

In other microbes, including *Legionella pneumophila* and *Acinetobacter baumannii*, the presence of plasmids is likewise not predictive of transformation capability (Mazzamurro et al., 2024). However, in *A. baumannii*, several prophage-associated genes have been negatively associated with transformability (Mazzamurro et al., 2024). Notably, in Gram-positive bacteria, certain plasmids and prophages can also actively inhibit competence; for example, plasmid-encoded ComI (Konkol et al., 2013) and *rok_LS20_* (Singh et al., 2012) block natural transformation in *B. subtilis,* while the prophage protein Prx can repress competence in streptococci (Mashburn-Warren et al., 2018). In our dataset, no statistically significant relationship was found between the number of prophages present per genome and transformation outcome (Welch’s two-sample *t*-test *p* = 0.79, Mann-Whitney *U* test *p* = 1.00). However, a modest trend was observed, with transformable isolates carrying fewer intact prophages on average (mean 1.27 per genome) than nontransformable isolates (mean 1.36). This is consistent with prior findings in *Streptococcus* and *Campylobacter jejuni*, where prophage–encoded DNases degrade extracellular DNA and inhibit natural transformation (Parker et al., 2024).

ComK is the master regulator of natural competence in *B. subtilis*, where it controls the expression of late competence genes and orchestrates the DNA‐uptake machinery (Dubnau & Losick, 2006). In *L. monocytogenes*, however, the *comK* locus is frequently disrupted by an A118-like prophage insertion. Consequently, the Com system is generally considered nonfunctional for mediating natural transformation and has instead been implicated in virulence modulation (Rabinovich et al., 2012). Nevertheless, because prophage-mediated disruption of *comK* would theoretically influence cellular physiology or DNA uptake capacity, we investigated whether *comK* status was associated with transformability in our dataset. As anticipated, no association was observed (Fisher's exact test *p* = 0.32); both intact and disrupted *comK* alleles were present among transformable (47 intact, 8 disrupted) and nontransformable (8 intact, 3 disrupted) isolates (Fig. 5).

**Figure 6:**
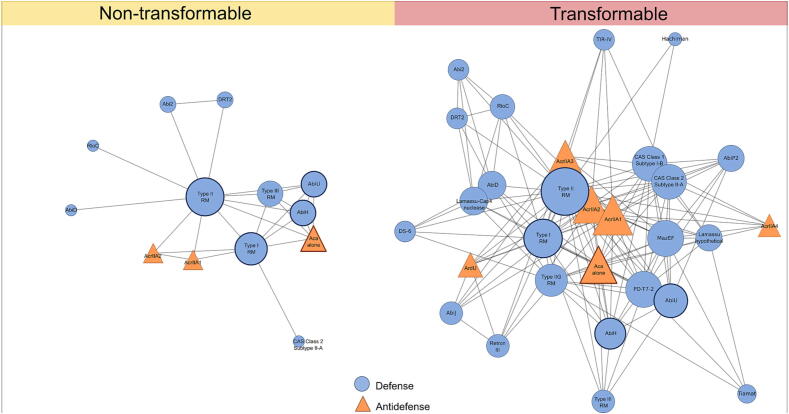
**Defense system network architecture in transformable vs. nontransformable *L. monocytogenes* isolates.** Isolates were classified as transformable or nontransformable based on experimental assessments of competence using the agar-lawn method. Cooccurrence networks were constructed using edges supported by ≥2 cooccurrence events. Node sizes reflect degree centrality (number of connections). Defense elements are represented as circles and antidefense elements as triangles. Features conserved in ≥80% of isolates are highlighted with bold outlines.

As components of bacterial immunity, defense systems such as RM and Abi protect cells from foreign DNA. RM enzymes distinguish self from non–self by methylating the host genome and cleaving unmethylated, exogenous sequences, while Abi modules detect phage infection and trigger programmed cell death to prevent viral replication (Stern and Sorek, 2011, Xu and Gu, 2024). Notably, defense systems such as Dodola, UG17, Retron I-C, and RosmerTA, along with antidefense elements including Dam and AcrIIA16, were detected exclusively in a subset of the nontransformable isolates (Fig. 5) from lineage I and lineage III. These elements, often associated with abortive infection or toxin-antitoxin mechanisms, may promote cell suicide or DNA degradation in response to foreign DNA (Bobonis et al., 2022, Millman et al., 2022, Sharifi and Ye, 2021, Wang et al., 2024), thereby inhibiting stable transformation. RM Type I and Type II, along with AbiH, AbiU, and Aca alone, were conserved in ≥80% of both transformable and nontransformable isolates (Fig. 6), reaffirming their status as core components of bacterial immunity (Makarova et al., 2020).

To test whether the cumulative burden of defense systems per genome was associated with transformability, we summed the number of all detected defense and antidefense loci per genome. Transformable and nontransformable isolates carried similar numbers of defense modules (5.74 ± 2.45 vs. 5.82 ± 2.18 systems per genome, respectively), with no significant differences detected by either Welch’s *t*-test (*p* = 0.92) or Mann–Whitney *U* test (*p* = 0.69). Analysis of per-system abundance showed no significant differences between transformable and nontransformable for most of the defense families, including RM, Cas, AbiD/U, retrons, and RloC (Supplementary Table 3). However, several systems displayed suggestive trends: Lamassu-like systems (Lamassu-Fam) were more abundant in transformable isolates, whereas anti-CRISPR and certain rare systems (All_UG, Dodola, RosmerTA) tended to occur exclusively or at higher copy number in nontransformable isolates (Supplementary Table 3). While the functional implications of this enrichment remain unclear, the presence of antidefense systems, often encoded by phages and other MGEs, can mediate competitive interactions and modulate the host compatibility of incoming MGEs (León et al., 2021, Maxwell, 2016, Pons et al., 2023).

To further investigate the relationship between defense and antidefense systems, we constructed a cooccurrence network. Network architecture varied between the two transformation phenotypes (Fig. 6). In nontransformable isolates, a sparse network of 13 defense systems with 26 edges (network density = 0.333, clustering coefficient = 0.322; Supplementary Table 4) was observed. In contrast, transformable isolates displayed a more interconnected network, with 29 defense systems forming 149 edges, a 5.7-fold increase in connectivity. Although network density was slightly higher (0.367), the average clustering coefficient was lower (0.221), suggesting greater connectivity but more distributed associations across subsystems. Despite these structural differences, bootstrap resampling (*n* = 10,000) found no significant differences in network density, clustering, or modularity between transformable and nontransformable groups (Supplementary Table 4). Taken together, the transformation capability in *L. monocytogenes* may be shaped by the diversity and interaction of its defense system repertoire. The expanded and more interconnected defense network observed in transformable isolates may reflect an adaptive immune strategy, enabling flexible responses to phage predation and other genomic threats. Future studies could investigate whether the defense and antidefense system patterns identified here remain stable under alternative growth or transformation conditions and whether these genomic features influence the success of transformation across additional isolate panels.

## Conclusion

In this study, we optimized transformation protocols for *L. monocytogenes* to overcome isolate-specific barriers and broaden the applicability of transformation methodologies across diverse genetic backgrounds. By increasing the voltage to 11 kV/cm and incorporating cAMP supplementation, we achieved dramatic improvements in competence with up to 377-fold increases compared to the original gold standard method. This enhancement was cAMP dose- and time-dependent, indicating a transient physiological shift potentially affecting membrane permeability or regulatory pathways distinct from natural competence. While the optimized gold standard protocol remains the most efficient method, we demonstrated that a simplified rapid agar-lawn approach offers a time-saving alternative, enabling transformation within three hours after the initial culture of input cells. Though less efficient, this method proved valuable for routine applications where throughput may be more important than yield. Our screen of 66 genetically diverse isolates using the simplified rapid agar-lawn approach revealed defense and antidefense system composition as a potential modulator of transformability. Overall, our findings lay the groundwork for more accessible and effective genetic manipulation of *L. monocytogenes*, a critical tool for functional genomics, pathogenesis studies, and synthetic biology. Future work should explore the mechanistic basis of cAMP-enhanced transformation and further investigate the relationship between MGEs and defense systems in shaping transformability across microbial populations.

## CRediT authorship contribution statement

**A.V. Gutiérrez:** Writing – review & editing, Writing – original draft, Visualization, Methodology, Investigation, Formal analysis, Data curation, Conceptualization. **N. Som:** Writing – review & editing, Investigation. **E. Smith:** Writing – review & editing, Investigation. **M. Diaz:** Writing – review & editing, Investigation, Data curation. **M. Matthews:** Writing – review & editing, Methodology, Investigation. **R.A. Kingsley:** Writing – review & editing, Conceptualization. **M. Gilmour:** Writing – review & editing, Conceptualization.

## Declaration of competing interest

The authors declare that they have no known competing financial interests or personal relationships that could have appeared to influence the work reported in this paper.
