# Supplementary material for: Rapid Preparation of Electrocompetent Listeria monocytogenes and Enhancement of Transformation Efficiency with cAMP Supplementation
Source: J Food Prot. 2026 May;89(5):100753. doi: 10.1016/j.jfp.2026.100753 (PMC13095671; doi:10.1016/j.jfp.2026.100753)
Supplement: Supplementary Data 1 [file mmc1.pptx]

## Slide 1
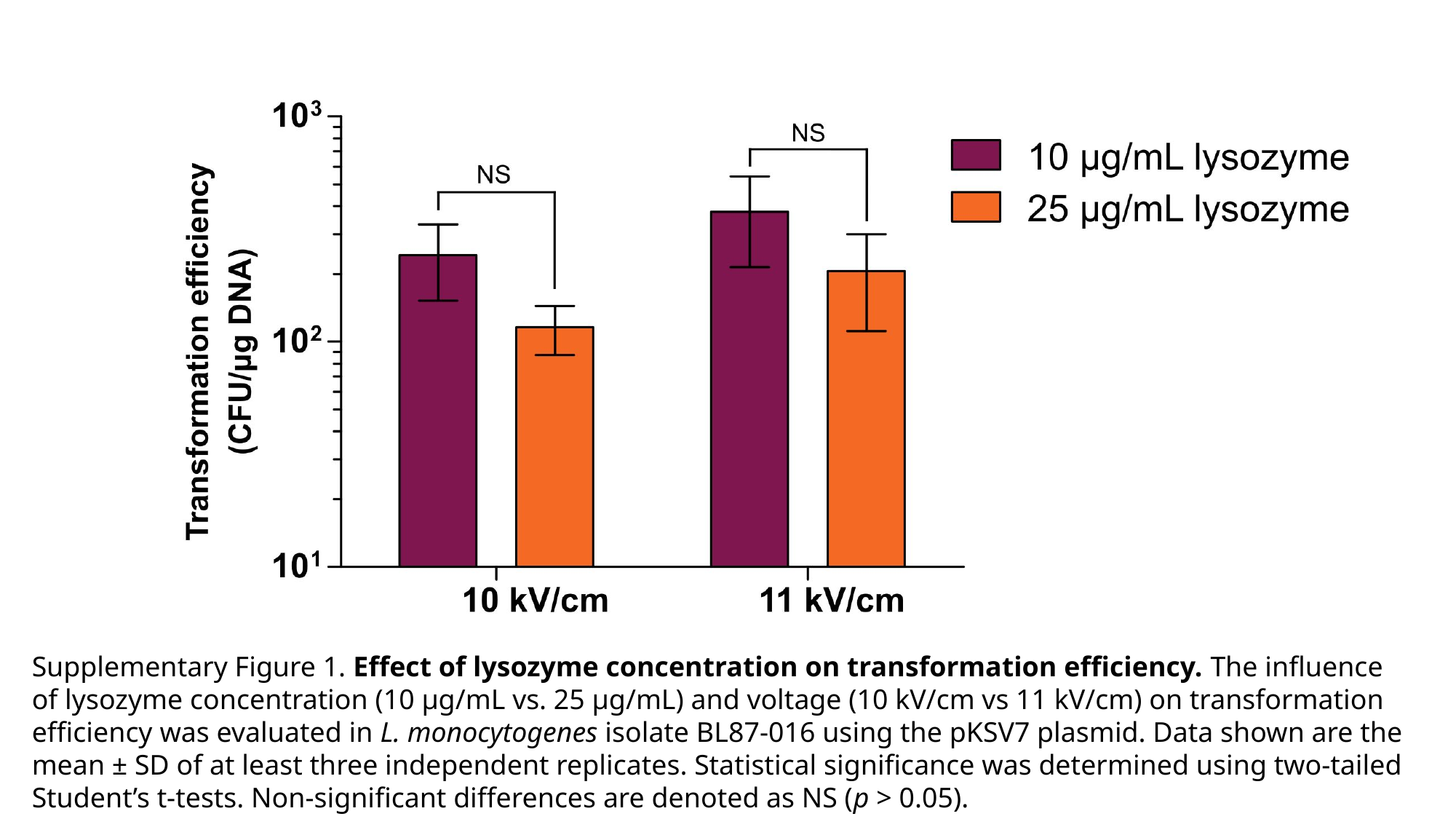

Supplementary Figure 1. Effect of lysozyme concentration on transformation efficiency. The influence of lysozyme concentration (10 μg/mL vs. 25 μg/mL) and voltage (10 kV/cm vs 11 kV/cm) on transformation efficiency was evaluated in L. monocytogenes isolate BL87-016 using the pKSV7 plasmid. Data shown are the mean ± SD of at least three independent replicates. Statistical significance was determined using two-tailed Student’s t-tests. Non-significant differences are denoted as NS (p > 0.05).

## Slide 2
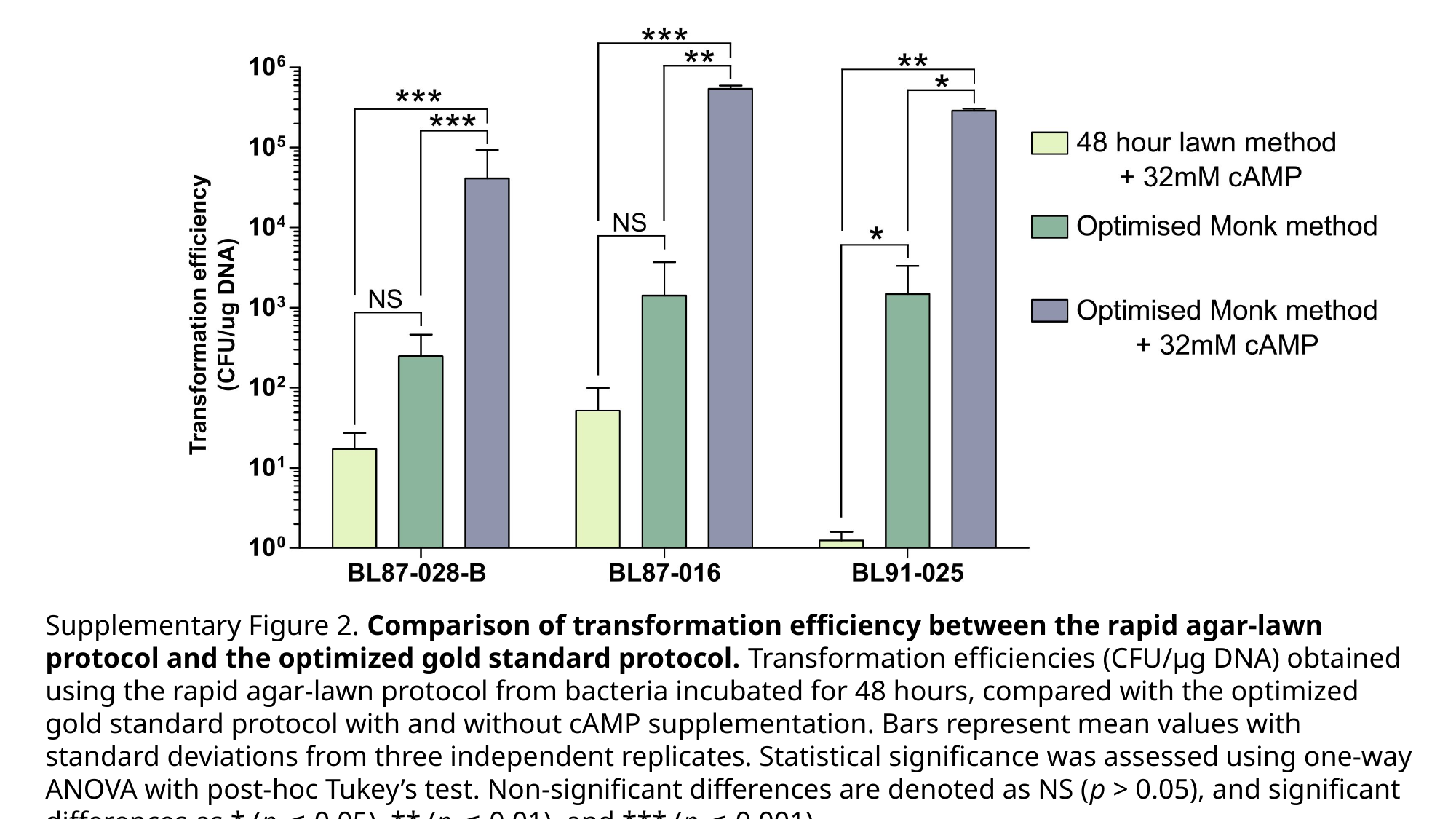

Supplementary Figure 2. Comparison of transformation efficiency between the rapid agar-lawn protocol and the optimized gold standard protocol. Transformation efficiencies (CFU/µg DNA) obtained using the rapid agar-lawn protocol from bacteria incubated for 48 hours, compared with the optimized gold standard protocol with and without cAMP supplementation. Bars represent mean values with standard deviations from three independent replicates. Statistical significance was assessed using one-way ANOVA with post-hoc Tukey’s test. Non-significant differences are denoted as NS (p > 0.05), and significant differences as * (p ≤ 0.05), ** (p ≤ 0.01), and *** (p ≤ 0.001).
